# From critical care units to postacute care facilities: the sooner, the better?

**DOI:** 10.62675/2965-2774.20240145-en

**Published:** 2024-08-07

**Authors:** Dimitri Gusmao-Flores, Bruna Brandão Barreto, Regis Goulart Rosa

**Affiliations:** 1 Hospital da Mulher Intensive Care Unit Salvador BA Brazil Intensive Care Unit, Hospital da Mulher - Salvador (BA), Brazil.; 2 Universidade Federal da Bahia Faculdade de Medicina da Bahia Salvador BA Brazil Postgraduate Program in Medicine and Health, Faculdade de Medicina da Bahia, Universidade Federal da Bahia - Salvador (BA), Brazil.; 3 Hospital Moinhos de Vento Internal Medicine Department Porto Alegre RS Brazil Internal Medicine Department, Hospital Moinhos de Vento - Porto Alegre (RS), Brazil.; 4 Brazilian Research in Intensive Care Network São Paulo SP Brazil Brazilian Research in Intensive Care Network (BRICNet) - São Paulo (SP), Brazil.

Since the 1990s, with a small interruption during the coronavirus disease 2019 (COVID-19) pandemic, admissions to postacute care facilities (PACFs) have been increasing progressively. Data obtained from the National Hospital Discharge Survey from 1996 to 2010 suggest a 49% relative increase in discharge to the PACF.^( 1 )^ Conversely, discharges to home declined by 5% over the same period. This trend seemed to be modified during the pandemic period of severe acute respiratory syndrome coronavirus 2 (SARS-CoV-2) infection, for several reasons that are not entirely clear. In this context, new data are necessary to clarify the dynamics of hospital discharge to the PACF, including the characteristics of transferred patients, the timing of transfers, patient locations (ward or intensive care unit [ICU]), and the trajectory of these patients within PACFs. This necessity is particularly pronounced in Brazil, where the scarcity of data on the feasibility and performance of PACFs adds a layer of complexity to understanding this transition.

Some of these research gaps were addressed by the retrospective cohort of Ramos et al., which included 847 patients admitted to a PACF directly from the ICU or intermediate care unit (IMCU) from 17 different hospitals in Brazil over a period of approximately 6 years.^( 2 )^ The main objective of the authors was to describe the trajectory functional recovery of those patients. All patients were evaluated using the Functional Independence Measure (FIM) upon admission and during follow-up and subsequently categorized as having no changes in functional status, worsening status, or improved functional status; most of them had rehabilitation as one of the criteria for admission, followed by palliative care. Approximately 35% of these patients were survivors of sepsis or stroke. Despite the fact that these patients were admitted directly from the ICU or IMCU and because a high readmission rate in acute hospitals could be expected, this occurred in only 20.4% of the patients. This frequency was less than that found in different cohorts with thousands of patients.^( 1 , 3 )^ Notwithstanding the distinct profile of the patients in these cohorts and the unclear location of these patients before discharge, it is acceptable to suppose that the majority were outside the ICU.

Another finding identified in the study by Ramos et al.^( 2 )^ was a high frequency of improving functionality in patients admitted to rehabilitation, measured with the FIM, during hospitalization in the PACF. Considering the negative impact of the loss of functional capacity in all aspects of the patient's life, including the return to work, observing a gain in functional status in 63% of the patients is significant. In a recent multicenter cohort, functional loss was the variable most strongly associated with not returning to work.^( 4 )^ The proportion of patients who improved functionally was quite superior to that in other studies, which could be explained by the profile of the patients (younger and with lower numbers of comorbidities) and/or by the program of rehabilitation offered in the PACF (up to 18 hours of therapy per week).

These findings are even more significant when considering patients discharged directly to home from the PACF. Compared to the FIM scores at admission, 83% of patients showed improvements in their functional capacities upon discharge to home. Consequently, almost 50% of patients improved their functional category, with a substantial decrease in total and severe functional dependence (from 84% in both categories at admission to 46% at discharge). However, improvement was not observed in patients admitted to palliative care.

Using the evaluation of functionality, Ramos et al.^( 2 )^ identified three trajectories in patients admitted to rehabilitation. More importantly, they managed to identify some characteristics that can help us identify patients who followed those paths, such as age, number of comorbidities, previous hospitalization, and use of enteral feeding and tracheostomy. Unfortunately, it is not possible to determine whether the disease leading to ICU admission also contributes to the different rehabilitation trajectories in this cohort. The evaluation of functionality is important because it helps us align expectations in the face of the uncertainty of recovery; moreover, it can help us identify patients who are more likely to benefit from an intensive rehabilitation program in a PACF during critical care.

Some questions remain unanswered. First, could the PACF be the preferred discharge destination for younger and "fitter" patients surviving critical illness? Most patients admitted to this institution and excluded from this cohort were from hospital wards (n = 1,175), and some of them were likely also survivors of critical illness. It would be interesting to compare the functional improvement of those transferred immediately from ICUs and those transferred after ward care. Second, what variables and clinical landmarks can help us identify patients who benefit from a modification of the goals of care during rehabilitation? Approximately 34% of patients admitted for rehabilitation had goals of care transitioned to palliative care, and although the majority of those changes occurred in the group with a deterioration of functional status, almost 20% of patients with an improvement of functionality also had goals of care changed to palliative care. As with time-limited trials, it would be interesting to estimate how long is long enough, and which variables could help us identify the optimal moment to realign goals of care to improve end-of-life outcomes and optimize resource use. Third, what happened with the cognitive trajectories? Although the FIM also evaluates cognitive function, in this cohort, the data are not presented separately, and cognitive impairment is also a relevant and prevalent disability after critical illness.^( 5 )^

In addition to decreasing mortality, intensive care physicians worldwide are also focused on how to decrease morbidity associated with post-ICU disabilities and improve the quality of life of survivors of critical illness. Simultaneous effort should be made not only in improving practices inside the ICU but also in identifying which group of patients benefit from different models of after-ICU care to accelerate functional recovery. The work by Ramos et al.^( 2 )^ suggested that discharge to the PACF directly from the ICU (or intermediate care units) in Brazil is feasible and potentially effective in improving functional outcomes compared with data from the literature; although this is still not enough to determine causality, it is an additional strategy to enhance recovery, aiming for functional independence upon returning home.
