# Supplementary material for: From critical care units to postacute care facilities: the sooner, the better?
Source: Crit Care Sci. 2024 Aug 7;36:e20240145en. doi: 10.62675/2965-2774.20240145-en (PMC11463977; doi:10.62675/2965-2774.20240145-en)
Supplement: Translation

# Translation: De unidades de terapia intensiva a unidades de cuidados pós-agudos: quanto mais cedo, melhor?

**DOI:** 10.62675/2965-2774.20240145-pt

**Authors:** Dimitri Gusmao-Flores, Bruna Brandão Barreto, Regis Goulart Rosa

**Affiliations:** 1 Salvador BA Brasil Unidade de Terapia Intensiva, Hospital da Mulher - Salvador (BA), Brasil.; 2 Salvador BA Brasil Programa de Pós-Graduação em Medicina e Saúde, Faculdade de Medicina da Bahia, Universidade Federal da Bahia - Salvador (BA), Brasil.; 3 Porto Alegre RS Brasil Departamento de Medicina Interna, Hospital Moinhos de Vento - Porto Alegre (RS), Brasil.; 4 São Paulo SP Brasil Brazilian Research in Intensive Care Network (BRICNet) - São Paulo (SP), Brasil.

Desde a década de 1990, com pequena interrupção durante a pandemia da doença causada pelo coronavírus 2019 (COVID-19), as internações em unidades de cuidados pós-agudos (PACFs - *post-acute care facilities* ) têm aumentado progressivamente. Dados obtidos da Pesquisa Nacional de Alta Hospitalar de 1996 a 2010 sugerem aumento relativo de 49% na alta para a PACF.^( 1 )^Por outro lado, as altas para casa diminuíram 5% no mesmo período. Essa tendência pareceu sofrer alterações durante o período pandêmico da infecção pelo coronavírus da síndrome respiratória aguda grave 2 (SARS-CoV-2), por vários motivos que não estão totalmente claros. Nesse contexto, são necessários novos dados para esclarecer a dinâmica da alta hospitalar para a PACF, incluindo as características dos pacientes transferidos, o momento das transferências, a localização dos pacientes (enfermaria ou unidade de terapia intensiva [UTI]) e a trajetória deles nas PACFs. Essa necessidade é particularmente acentuada no Brasil, onde a escassez de dados sobre a viabilidade e o desempenho das PACFs confere mais complexidade à compreensão dessa transição.

Algumas dessas lacunas de pesquisa foram abordadas pela coorte retrospectiva de Ramos et al., que incluiu 847 pacientes admitidos em uma PACF diretamente da UTI ou da unidade de cuidados intermediários (UCI) de 17 hospitais diferentes no Brasil durante um período de aproximadamente 6 anos.^( 2 )^O principal objetivo dos autores foi descrever a trajetória de recuperação funcional desses pacientes. Todos os pacientes foram avaliados usando a Medida de Independência Funcional (MIF) na admissão e durante o seguimento e, posteriormente, categorizados como sem alterações no estado funcional, com piora ou com melhora do estado funcional; a maioria deles tinha a reabilitação como um dos critérios de admissão, seguida de cuidados paliativos. Aproximadamente 35% desses pacientes eram sobreviventes de sepse ou acidente vascular cerebral. Apesar do fato de esses pacientes terem sido admitidos diretamente da UTI ou da UCI e de ser esperada uma alta taxa de readmissão em hospitais de agudos, isso ocorreu em apenas 20,4% dos pacientes. Essa frequência foi menor do que a encontrada em diferentes coortes com milhares de pacientes.^( 1 , 3 )^Apesar do perfil distinto dos pacientes nessas coortes e da localização pouco clara desses pacientes antes da alta, é aceitável supor que a maioria estava fora da UTI.

Outro achado identificado no estudo de Ramos et al.^( 2 )^foi a alta frequência de melhora da funcionalidade em pacientes admitidos para reabilitação, medida com a MIF, durante a internação na PACF. Considerando o efeito negativo da perda da capacidade funcional em todos os aspectos da vida do paciente, incluindo o retorno ao trabalho, é significativo observar ganho no estado funcional em 63% dos pacientes. Em uma coorte multicêntrica recente, a perda funcional foi a variável mais fortemente associada ao não retorno ao trabalho.^( 4 )^A proporção de pacientes que melhoraram funcionalmente foi bastante superior à de outros estudos, o que se explicaria pelo perfil dos pacientes (mais jovens e com menor número de comorbidades) e/ou pelo programa de reabilitação oferecido na PACF (até 18 horas de terapia por semana).

Esses achados são ainda mais significativos quando se consideram os pacientes que receberam alta da PACF diretamente para casa. Em comparação com os escores da MIF na admissão, 83% dos pacientes apresentaram melhora em suas capacidades funcionais na alta para casa. Consequentemente, quase 50% dos pacientes melhoraram sua categoria funcional, com redução substancial na dependência funcional total e grave (de 84% em ambas as categorias na admissão para 46% na alta). No entanto, não houve melhora nos pacientes admitidos para cuidados paliativos.

Ramos et al.^( 2 )^identificaram três trajetórias em pacientes admitidos para reabilitação usando a avaliação da funcionalidade. Mais importante ainda, eles conseguiram identificar algumas características que podem nos ajudar a identificar os pacientes que seguiram essas trajetórias, como idade, número de comorbidades, hospitalização anterior e uso de alimentação enteral e traqueostomia. Infelizmente, não é possível determinar se a doença que levou à internação na UTI também contribui para as diferentes trajetórias de reabilitação nessa coorte. A avaliação da funcionalidade é importante, porque nos ajuda a alinhar as expectativas diante da incerteza da recuperação; além disso, pode nos ajudar a identificar os pacientes com maior probabilidade de se beneficiar de um programa de reabilitação intensivo em uma PACF durante o tratamento crítico.

Algumas perguntas continuam sem resposta. Em primeiro lugar, a PACF poderia ser o destino preferido de alta para pacientes mais jovens e "em forma" que sobreviveram a doenças graves? A maioria dos pacientes admitidos nessa instituição e excluídos dessa coorte era de enfermarias hospitalares (n = 1.175), e é provável que alguns deles também tenham sobrevivido a doenças graves. Seria interessante comparar a melhora funcional daqueles que foram transferidos imediatamente das UTIs e daqueles que foram transferidos após os cuidados na enfermaria. Em segundo lugar, quais variáveis e marcos clínicos podem nos ajudar a identificar os pacientes que se beneficiam de uma alteração das metas de tratamento durante a reabilitação? Aproximadamente 34% dos pacientes admitidos para reabilitação tiveram as metas de tratamento transferidas para cuidados paliativos e, embora a maioria dessas mudanças tenha ocorrido no grupo com deterioração do estado funcional, quase 20% dos pacientes com melhora da funcionalidade também tiveram as metas de tratamento alteradas para cuidados paliativos. Assim como acontece com os estudos de tempo limitado, seria interessante estimar quanto tempo é suficiente e quais variáveis poderiam nos ajudar a identificar o momento ideal para realinhar as metas de tratamento para melhorar os desfechos no final da vida e otimizar o uso de recursos. Terceiro, o que aconteceu com as trajetórias cognitivas? Embora a MIF também avalie a função cognitiva, nessa coorte os dados não são apresentados separadamente, e o comprometimento cognitivo também é uma deficiência relevante e prevalente após uma doença crítica.^( 5 )^

Além de diminuir a mortalidade, os médicos intensivistas de todo o mundo também estão concentrados em como diminuir a morbidade associada às deficiências pós-UTI e melhorar a qualidade de vida dos sobreviventes de doenças graves. Deve-se fazer um esforço simultâneo não apenas para melhorar as práticas dentro da UTI, mas também para identificar qual grupo de pacientes se beneficia de diferentes modelos de cuidados pós-UTI para acelerar a recuperação funcional. Ramos et al.^( 2 )^sugeriram que a alta para a PACF diretamente da UTI ou das UCIs no Brasil é viável e potencialmente eficaz na melhoria dos desfechos funcionais em comparação com os dados da literatura; embora isso ainda não seja suficiente para determinar a causalidade, é uma estratégia adicional para melhorar a recuperação, visando à independência funcional no retorno para casa.
